# Evaluation of AISI Type 304 stainless steel as a suitable surface material for evaluating the efficacy of peracetic acid-based disinfectants against *Clostridium difficile* spores

**DOI:** 10.1371/journal.pone.0187074

**Published:** 2017-10-24

**Authors:** Elaine Black, Krista Owens, Richard Staub, Junzhong Li, Kristen Mills, Justin Valenstein, John Hilgren

**Affiliations:** Ecolab Research, Development and Engineering Center, Eagan, Minnesota, United States of America; University of Connecticut, UNITED STATES

## Abstract

Disinfectants play an important role in controlling microbial contamination on hard surfaces in hospitals. The effectiveness of disinfectants in real life can be predicted by laboratory tests that measure killing of microbes on carriers. The modified Quantitative Disk Carrier Test (QCT-2) is a standard laboratory method that employs American Iron and Steel Institute (AISI) Type 430 stainless steel carriers to measure hospital disinfectant efficacy against *Clostridium difficile* spores. The formation of a rust-colored precipitate was observed on Type 430 carriers when testing a peracetic acid (PAA)-based disinfectant with the QCT-2 method. It was hypothesized that the precipitate was indicative of corrosion of the Type 430 carrier, and that corrosion could impact efficacy results. The objective of this study was to compare the suitability of AISI Type 430 to Type 304 stainless steel carriers for evaluating PAA-based disinfectants using the QCT-2 method. Type 304 is more corrosion-resistant than Type 430, is ubiquitous in healthcare environments, and is used in other standard methods. Suitability of the carriers was evaluated by comparing their impacts on efficacy results and PAA degradation rates. In efficacy tests with 1376 ppm PAA, reductions of *C*. *difficile* spores after 5, 7 and 10 minutes on Type 430 carriers were at least about 1.5 log_10_ lower than reductions on Type 304 carriers. In conditions simulating a QCT-2 test, PAA concentration with Type 430 carriers was reduced by approximately 80% in 10 minutes, whereas PAA concentration in the presence of Type 304 carriers remained stable. Elemental analyses of residues on each carrier type after efficacy testing were indicative of corrosion on the Type 430 carrier. Use of Type 430 stainless steel carriers for measuring the efficacy of PAA-based disinfectants should be avoided as it can lead to an underestimation of real life sporicidal efficacy. Type 304 stainless steel carriers are recommended as a suitable alternative.

## Introduction

Disinfectants are used in hospitals, long-term care facilities, restaurants, hotels and homes to kill microorganisms that can cause serious illnesses [1]. Regulatory authorities rely on the use of standard laboratory methods to predict the effectiveness of disinfectants under real life conditions. For example, the United States Environmental Protection Agency (EPA) and the European Chemicals Agency (ECHA) require data from standard efficacy methods to regulate disinfectant products claiming to kill microbes of public health importance [2, 3]. Generally, standard efficacy methods are developed and maintained by non-governmental organizations such as ASTM International, AOAC International, the European Committee for Standardization (CEN, French: Comité Européen de Normalisation) and the Organisation for Economic Cooperation and Development (OECD).

Disinfectant efficacy may be evaluated by what are known as suspension or carrier methods. The basic steps of suspension methods are as follows: (i) preparation of the microbe test suspension, (ii) exposure to the disinfectant, (iii) neutralization or removal of the active antimicrobial agent, and (iv) enumeration of the surviving population. Suspension tests are useful in the early stages of disinfectant product development to understand kill kinetics. The basic steps of carrier methods are the same as suspension methods except that the microbe test suspension is dried onto a carrier before exposure to the disinfectant. Carrier tests are useful for predicting the efficacy of disinfectants in specific, real life uses. Carriers used in testing can differ in size, shape, and material of construction. The two most common carrier materials used in standard disinfection methods are glass and AISI Type 304 austenitic stainless steel (Table 1).

**Table 1:** Standard antimicrobial test methods and carrier types.

Method	Title	Carrier Type(s)
EN 13697	Quantitative non-porous surface test for the evaluation of bactericidal or fungicidal activity	AISI Type 304 stainless steel with No.2B finish on both sides
EN 14561	Chemical disinfectants and antiseptics—Quantitative carrier test for the evaluation of bactericidal activity for instruments used inthe medical area	Glass
AOAC 960.09	Germicidal and Detergent Sanitizing Action of Disinfectants	Polished stainless steel cylinders; AISI Type 304 stainless steel
AOAC 961.02	Germicidal Spray Products as Disinfectants	Glass carriers
ASTM 1153–14	Standard Test Method for Efficacy of Sanitizers Recommended for Inanimate, Hard, Nonporous Non-Food Contact Surfaces	Carrier of ‘appropriate type’–glass and AISI Type 304 stainless steel commonly used
AOAC 966.04	Sporicidal Activity of Disinfectants Test	Porcelain Penicylinders, silk or Dacron® suture loops
ASTM E2871-13	Standard Test Method for Evaluating Disinfectant Efficacy against *Pseudomonas aeruginosa* Biofilm Grown in CDC Biofilm Reactor Using Single Tube Method	Borosilicate glass
ASTM 2197–11	Standard Quantitative Disk Carrier Test Method for Determining the Bactericidal, Virucidal, Fungicidal, Mycobactericidal and Sporicidal Activities of Liquid Chemical Germicides	AISI Type 430 stainless steel with No.4 finish on one side

The relevance of the carrier material to the intended use-sites should be considered to ensure method utility [4]. Allowances for alternative carrier materials have been made when it has been determined that the material specified in the standard method is not a suitable predictor of real life efficacy. For example, Dacron® suture material was proposed as an alternative to silk suture material for evaluating the sporicidal efficacy of PAA-based disinfectants agents because silk caused PAA degradation [5, 6]. As a result of this research, AOAC International and the EPA accept the use of Dacron®, instead of silk, as a carrier material for PAA-based disinfectants for the AOAC Sporicidal Activity Test [7, 8]. An additional element that can be incorporated into efficacy testing is the addition of interfering substances to the bacterial inoculum to represent real life challenges to disinfection efficacy caused by blood or other soils [9, 10, 11]. For example, blood serum, mucin and yeast extract are used as interfering substances in the QCT-2 method [12].

Development of disinfectants to help control *Clostridium difficile* infections is critically important. *C*. *difficile* is the most common cause of health care–associated infections in U.S. hospitals. The health care costs related to *C*. *difficile* infection are estimated to be as much as $4.8 billion for acute care facilities [13]. In healthcare settings, the surfaces of commodes and bathing tubs are known to become contaminated with feces and serve as a reservoir for *C*. *difficile* spores. *C*. *difficile* spores are transferred to patients mainly via the hands of healthcare personnel who have touched a contaminated surface or item [14]. Thus, it is important for carriers in *C*. *difficile* disinfectant efficacy tests to be representative of the hard surfaces in healthcare environments. It is also important to consider chemical compatibility of the surface material because, in real life, hospital surfaces need to withstand frequent exposures to commercial detergents and disinfectants. Glass and certain types of stainless steel have been used as carrier materials because of their good chemical compatibility.

The QCT-2 test method for *C*. *difficile* efficacy specifies that carriers be constructed of a ferritic (magnetic) grade of stainless steel known as AISI Type 430. The magnetic nature of this material provides ease of handling during laboratory testing; however, a potential problem with Type 430 stainless steel is that it is more likely to corrode in the presence of certain chemicals. For example, expert guidelines recommend that Type 430 be used for decorative applications in mild atmospheres [15]. In contrast, Type 304 stainless steel is used extensively in kitchen and hospital equipment and resists organic chemicals and a wide variety of inorganic chemicals. When evaluating stainless steels for potential compatibility with PAA-based disinfectants, it is important to consider that PAA is always present with acetic acid and hydrogen peroxide. Ferritic stainless steels such as Type 430 are not endorsed for use with acetic acid. In contrast, Type 304 is endorsed for use with acetic acid [16].

A rust-colored precipitate was observed on Type 430 stainless steel carriers during C. difficile sporicidal efficacy testing of a PAA-based disinfectant using the QCT-2 method. It was hypothesized that the precipitate formed following an oxidation reaction of PAA-based disinfectant with iron from the stainless steel, producing iron oxides. This reaction is significantly enhanced by a relatively high level of chloride ions present. Chloride ions from the spore inoculum originate from phosphate buffered saline (PBS; 0.8% NaCl) used as the spore and interfering substances diluent. There is approximately 0.048 mg of chloride deposited on a carrier in 10 μL of spore inoculum in the QCT-2 test. In a healthcare environment, the maximum amount of chloride estimated to be present from 10 μL of feces is much lower; we estimate 0.014 mg based on an established reference range for chloride in feces [17].

Based on these considerations, studies were initiated to understand if the rust-colored precipitate observed on Type 430 carriers after exposure to a PAA-based disinfectant in QCT-2 testing was associated with PAA degradation. Such method-specific degradation could result in an underestimation of the PAA product’s efficacy under real life healthcare conditions and uses. First, as a baseline, the *C*. *difficile* spore-killing performance of a PAA-based disinfectant was characterized in a suspension test where carrier type was not a factor. Then, a comparison of AISI Type 430 to AISI Type 304 stainless steel carriers was made in three ways: (i) their impact on PAA degradation rate, (ii) their impact on efficacy in the QCT-2 test, and (iii) their susceptibility to corrosion based on elemental analysis after exposure to PAA in the QCT-2 test. Type 304 stainless steel was chosen for comparison because of its better resistance to corrosion, ubiquity as a material in medical and pharmaceutical equipment, and history as a suitable carrier in AOAC and CEN standard methods.

## Materials and methods

### Bacterial strain and spore preparation and purification

The strain used throughout this study was *C*. *difficile* ATCC 43598 (American Type Culture Collection, Manassas, VA) because it is the strain specified in the QCT-2 method to support EPA registration. A spore suspension was prepared following the EPA standard operating procedure [18]. In brief, a frozen stock culture was grown on plates of CDC anaerobic 5% sheep blood agar (CABA; Anaerobe Systems, Morgan Hill, CA) for 10 days. After harvesting, the spore suspension was purified using 50% (w/v) solution of HistoDenz (Sigma Aldrich, St Louis, MO). The final spore preparation was suspended in Phosphate Buffered Saline + 0.1% tween80 (PBS-T) and stored at -80°C until used.

### Interfering substance preparation

The interfering substance was prepared according to the QCT-2 method described previously. In brief, three stock components were made separately and stored at -20°C until used; (i) 0.5 g of bovine serum albumin (BSA; EMD Millipore, Billerica, MA) in 10 mL of phosphate buffered saline (PBS), filtered with a 0.2 μm pore diameter membrane filter, (ii) 0.5 g of yeast extract (Oxoid, Basingstoke, UK) in 10 mL of PBS, filtered with a 0.2 μm pore filter, and (iii) 0.04 g of bovine mucin (MP Biomedicals, Santa Ana, CA) in 10 mL of PBS, autoclaved (15 minutes at 121°C). A final spore suspension containing all three interfering substances was prepared by combining 25 μL of BSA stock, 35 μL of yeast extract stock, 100 μL of mucin stock and 340 μL of final spore suspension.

### Peracetic acid-based disinfectant working solution preparation

A commercially available PAA-based disinfectant product (Ecolab, Inc., Saint Paul, MN) was used in all experiments. Working solutions containing approximately 1300 parts per million (ppm) by weight PAA were prepared by diluting a concentrated PAA-based product in 400 ppm (as CaCO_3_) AOAC Synthetic Hard Water [19]. All working solutions were used within 2 hours of preparation.

### Suspension efficacy test

Using aseptic technique 100 μL quantities of the spore suspension containing interfering substances were added to separate micro-centrifuge tubes containing 500 μL of the PAA working solution (resulting in a final PAA concentration of approximately 1100 ppm in each micro-centrifuge tube). This ratio was selected to mimic the spore-to-chemical ratio utilized in carrier tests. At exposure times of 1, 3, 5, 7 and 10 minutes, 100 μL of the contents of one tube was transferred to 10 mL of neutralizer (PBS + 0.1% Tween®80 + 0.5% sodium thiosulfate). Spores that survived the treatment were enumerated by spread-plating 0.1 mL and 1.0 mL quantities of the neutralizer on Brain Heart Infusion agar with horse blood and taurocholate (BHIY-HT; Anaerobe Systems, Morgan Hill, CA). In addition, the remainder of the neutralizer was membrane filtered and plated. An initial population control was performed by substituting PBS-T for the PAA working solution, transferring 100 uL of this mixture to 10 mL of PBS-T and spread-plating a set of serial dilutions from this tube to BHIY-HT. All plates were incubated anaerobically at 35°C for 5 days prior to enumeration.

### Carrier efficacy test

Sporicidal efficacy on Types 430 and 304 stainless steel carriers (Pegen Industries, Ontario Canada) was carried-out according to the QCT-2 method. One independent test examined 5 and 7 minute exposure times. A separate independent test examined a 10 minute exposure time. For each test, at least 5 treated carriers and 3 control carriers were evaluated. The neutralizer was verified to be effective prior to testing. For dilution and recovery of treated and control spores for either stainless steel type, no magnet was used to hold carriers to the bottoms of the vials while pouring the contents into a filter unit; the carrier remained in the vial due to adhesive forces between the liquid and the vial bottom while pouring.

### Peracetic acid degradation test

Carriers of Types 304 and 430 were inoculated as described previously in the carrier efficacy test, except replacing the spore suspension with only sterile PBS-T. Five (5) mL of the PAA working solution was then added to a flask containing 50 carriers of each type. This equated to 100 μL of PAA working solution per carrier. Samples from the flasks were pulled at times 0, 1, 3, 5, 10 and 15 minutes after addition and tested for PAA by iodometric titration [20]. Titrations of the PAA solution, without carrier interference, analyzed at the time intervals above were used as a control.

### Residues on carrier surfaces after simulated efficacy testing

Carriers of Types 304 and 430 stainless steel were prepared in a ‘simulated efficacy test’ i.e. all reagents used for efficacy testing (PBS, interfering substances and PAA disinfectant) were applied to coupons without addition of spores, neutralized at 10 mins and allowed to dry. Residues remaining on the surfaces of Types 304 and 430 carriers were analyzed by Energy Dispersive X-Ray Spectroscopy. A residue sample was transferred to an adhesive carbon tape on a specimen holder. The samples were bombarded with a 15 keV electron beam source from a Hitachi S3400N scanning electron microscope and subsequently emitted x-rays from the sample were detected by a Thermo Scientific UltraDry detector to characterize the elements present in the sample.

## Results

### Suspension test

**Fig 1:**
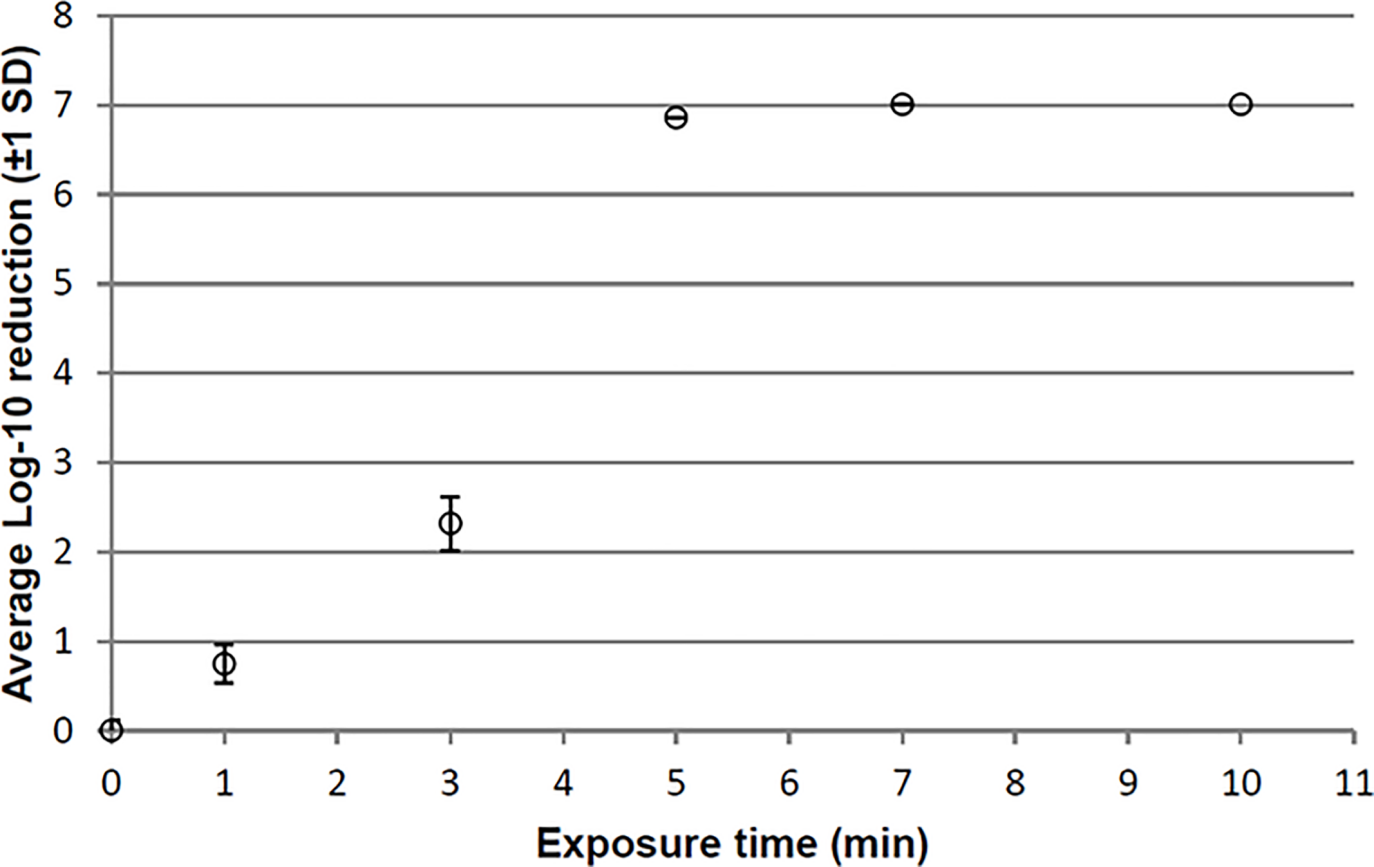
Suspension test: Reductions of *C*. *difficile* spores in 1147 ppm peracetic acid (starting concentration) at 20°C.

A plot of *C*. *difficile* spore kill over time at 20°C revealed a sigmoidal curve (Fig 1). The inflection point of the curve occurred in the 3–4 minute range, with maximum *C*. *difficile* spore reductions beginning after approximately 5 minutes. Spore kill after 7 and 10 minutes was not obviously different than 5 minutes. Based on these results, we selected to test efficacy after 5, 7 and 10 minutes in carrier efficacy tests, assuming the killing of spores dried onto a carrier would be more challenging than killing spores in liquid suspension.

### Carrier test

Efficacy testing of *C*. *difficile* spores dried onto stainless steel carriers resulted in obvious differences in log reductions between Types 430 and 304 stainless steels (Table 2). Specifically, at least 6-log_10_ reductions were observed on Type 304 carriers after 5, 7 and 10 minutes; however, less than approximately 4.5-log reductions were observed on Type 430 carriers at the same time points. A rust-colored precipitate was observed on the surfaces of Type 430 carriers after 10 minutes. The discoloration was localized to the inoculation site on the carrier. No discoloration was observed on Type 304 carriers after 10 minutes.

**Table 2:** Carrier test: Reductions of *C*. *difficile* spores by 1376 ppm peracetic acid (starting concentration) at 20°C.

Exposure time (min)	Log_10_ reductionᵃ (and standard deviation)	
	AISI Type 430 stainless steel	AISI Type 304 stainless steel
5	≤4.48ᵇ	6.86^c^
7	≤4.44ᵇ	6.59 (0.24)
10	≤4.56ᵇ	6.84^c^

ᵃLog_10_ reduction = mean log_10_ control—mean log_10_ treatment

ᵇThe number of colony forming units on all plates, including those from the highest dilution of the treatment eluate, exceeded 200. No standard deviation calculated.

^c^The number of colony forming units on all plates was zero. No standard deviation calculated.

### Peracetic acid degradation test

**Fig 2:**
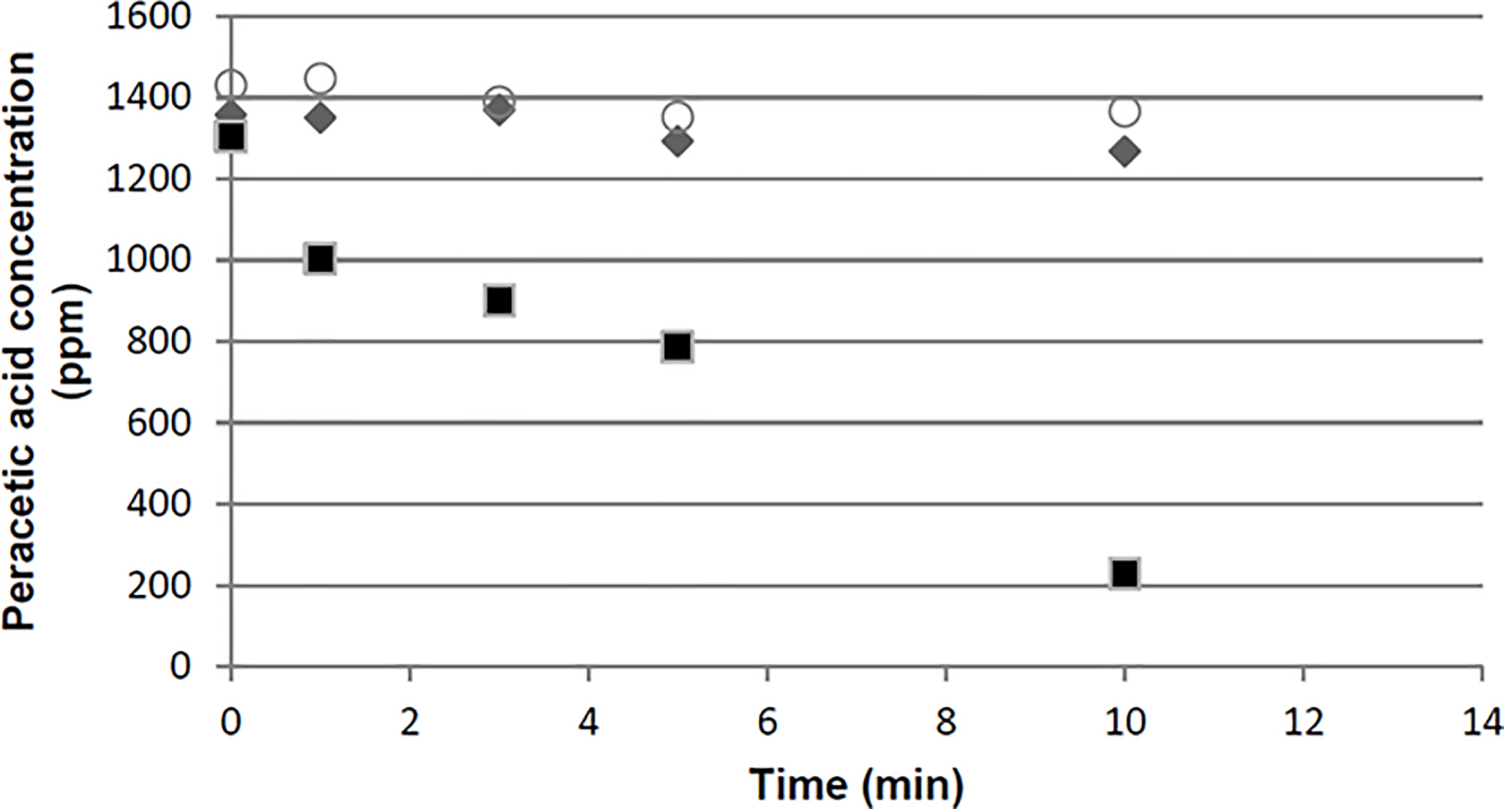
Changes in peracetic acid concentration when exposed to AISI types 304 (◆) or AISI 430 (■) stainless steel carriers containing dried-on phosphate buffered saline. PAA concentration at 0, 1, 3, 5, 10 and 15 minutes provide control (○) points.

The concentration of PAA declined substantially over time in the test with Type 430 carriers (Fig 2). Specifically, after 5 and 10 minutes, the concentrations of PAA in the presence of Type 430 carriers were 787 ppm (60% of starting) and 227 ppm (17% of starting), respectively. In contrast, the concentrations of PAA after 5 and 10 minutes in the presence of Type 304 carriers were 1292 ppm (95% of starting) and 1267 ppm (93% of starting), respectively. In this test there was 100 μL of PAA working solution per carrier whereas there was 50 μL of PAA working solution per carrier in the efficacy test. Thus, the results of the PAA degradation test provide directionally useful information, but may not represent the true PAA degradation rate in a carrier test. When one considers that maximum efficacy of the PAA working solution requires at least about 5 minutes (estimated from the suspension test results), and that about 40% of the PAA is lost by 5 minutes in a carrier test with Type 430 carriers (versus virtually no loss with Type 304 carriers), then it is not surprising why there are obvious differences in carrier efficacy test results.

### Analysis of residual soil after simulated efficacy test

**Fig 3:**
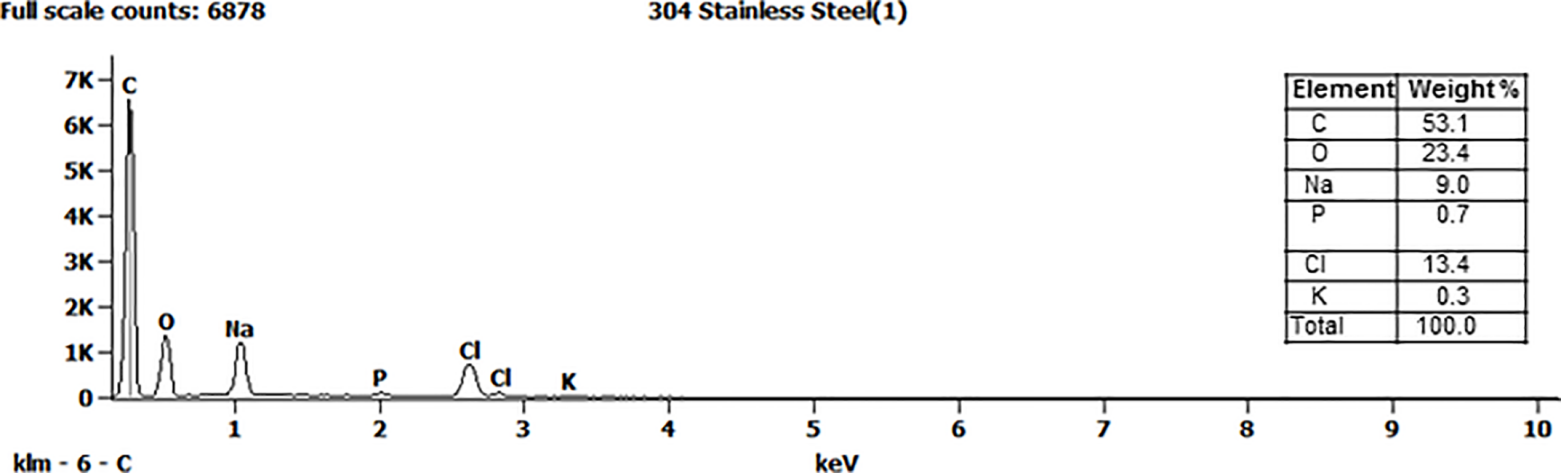
Energy dispersive X-ray spectroscopy: elements in the residue on an AISI Type 304 carrier after efficacy testing with the QCT-2 method.

**Fig 4:**
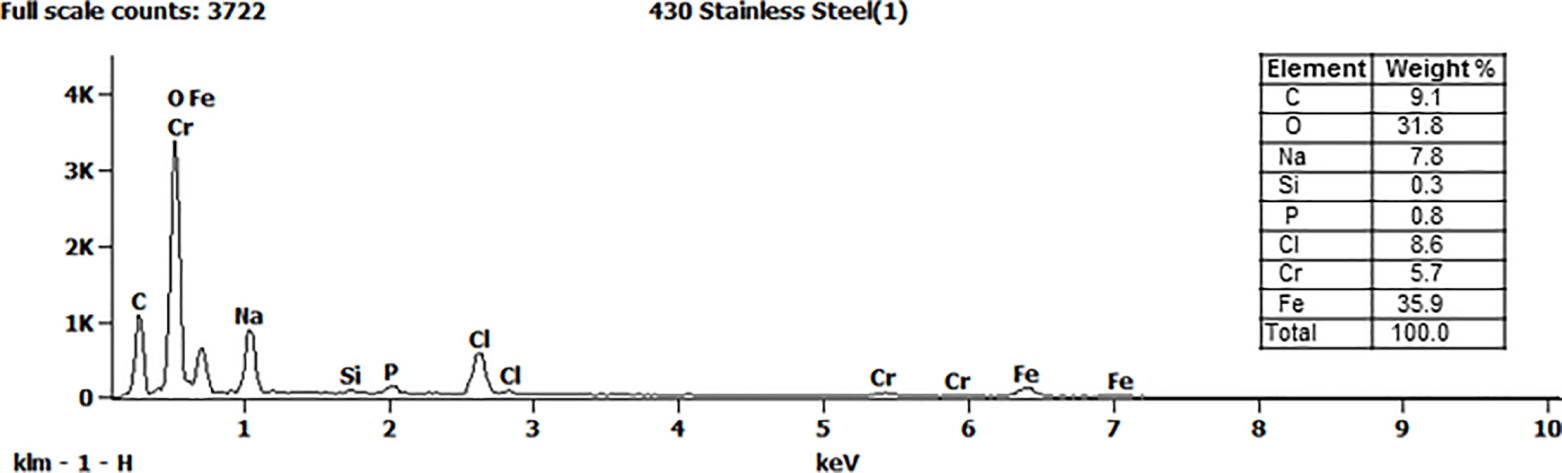
Energy dispersive X-ray spectroscopy: elements in the residue on an AISI Type 430 carrier after efficacy testing with the QCT-2 method.

The elements detected on the surface of a Type 304 carrier were carbon, oxygen, sodium, phosphorus, chloride and potassium (Fig 3). All these elements were consistent with those known to be present in the QCT-reagents and interfering substance. In contrast, elements present on the surface of the Type 430 carrier included two elements not detected on the Type 304 carrier: chromium and iron (Fig 4). The presence of the chromium and iron are indicative of corrosion on the carrier surface.

## Discussion

The test conditions and materials used in laboratory efficacy methods should be representative of actual uses; otherwise they may not be good predictors of real life results. The results of this study raise questions about the suitability of Type 430 stainless steel carriers to predict the efficacy of PAA-based disinfectants in healthcare environments.

AISI Type 430 stainless steel is essentially nickel free, and is consequently less resistant to corrosion than those alloyed with nickel, such as AISI Type 304. While Type 430 is a common ferritic grade of stainless used in modern industry [21], its usefulness is limited to objects and fittings that do not require robust resistance to corrosion such as low-grade cutlery, work surfaces and cabinets, and trim on domestic cookers [22, 23, 24]. Although Type 430 stainless steel provides convenience in the QCT-2 test because of its magnetic properties, it is not relevant to hospital use sites because it does not possess the level of corrosion resistance required for environments that are disinfected frequently [21].

It is vital to have a test method that accurately measures the efficacy of PAA-based disinfectants. PAA-based disinfectants are used extensively in the food and healthcare industries [25]. PAA-based disinfectants may be preferred over other disinfectants because PAA decomposes into benign substances (acetic acid and oxygen) and provides excellent disinfection in a short time period.

Using the QCT-2 method with Type 430 stainless steel carriers could lead to unnecessary over-formulation of PAA-based disinfectants because the level of PAA in these products would need to be higher to compensate for degradation; an artifact from using Type 430 stainless steel. Over-formulation has negative safety and economic consequences because of needless use of, and exposure to higher to, PAA and other chemicals.

## Conclusion

AISI Type 430 stainless steel is not a suitable carrier material for laboratory efficacy tests to predict the real life efficacy of PAA-based disinfectants in healthcare settings. The authors recommend AISI Type 304 stainless steel as an acceptable alternative carrier material for efficacy testing of PAA-based disinfectants.
